# Psychosis, vulnerability, and the moral significance of biomedical innovation in psychiatry. Why ethicists should join efforts

**DOI:** 10.1007/s11019-019-09932-4

**Published:** 2019-11-26

**Authors:** Paolo Corsico

**Affiliations:** Centre for Social Ethics and Policy, Department of Law, School of Social Sciences, The University of Manchester, Williamson Building, Oxford Road, Manchester, M13 9PL UK

**Keywords:** Genomics, Neuroscience, Psychosis, Technological convergence, Vulnerability

## Abstract

The study of the neuroscience and genomics of mental illness are increasingly intertwined. This is mostly due to the translation of medical technologies into psychiatry and to technological convergence. This article focuses on psychosis. I argue that the convergence of neuroscience and genomics in the context of psychosis is morally problematic, and that ethics scholarship should go beyond the identification of a number of ethical, legal, and social issues. My argument is composed of two strands. First, I argue that we should respond to technological convergence by developing an integrated, patient-centred approach focused on the assessment of individual vulnerabilities. Responding to technological convergence requires that we (i) integrate insights from several areas of ethics, (ii) translate bioethical principles into the mental health context, and (iii) proactively try to anticipate future ethical concerns. Second, I argue that a nuanced understanding of the concept of vulnerability might help us to accomplish this task. I borrow Florencia Luna’s notion of ‘layers of vulnerability’ to show how potential harms or wrongs to individuals who experience psychosis can be conceptualised as stemming from different sources, or layers, of vulnerability. I argue that a layered notion of vulnerability might serve as a common ground to achieve the ethical integration needed to ensure that biomedical innovation can truly benefit, and not harm, individuals who suffer from psychosis.

## Introduction

Bioethics and mental health often had a difficult or at least uneasy relationship (Holm 2019). Whilst bioethics has evolved into an interdisciplinary discourse around the ethical implications of technological innovation in biomedicine, mental health ethics has largely been dominated by issues of capacity, coercion, and involuntary hospitalisation of the mentally ill (Sadler et al. 2015). In this article I argue that the relationship between bioethics and mental health should, to some extent, be revised. An epistemological shift in the way we understand this relationship is required by the historical occurrences which have brought some of the hardest strands of biomedicine—neuroscience and genomics—to play a crucial role in the efforts to unveil the nature of mental illness and to find ways to prevent it, and cure it.

This article focuses on *psychosis*. The past few decades have seen a rapid increase in the use of neuroscience and genomics to investigate psychosis and susceptibility to psychotic disorders. On the one hand, neuroimaging studies are shedding light on the cognitive and neurobiological correlates of psychosis (Fusar-Poli et al. 2012). On the other hand, large cohort Genome-Wide Association Studies (GWAS) are playing an important role in the investigation of the genomic basis of psychosis, while whole genome sequencing is increasingly used to investigate susceptibility to psychotic illness (Schizophrenia Working Group of the Psychiatric Genomics Consortium 2014). More importantly, the study of the neuroscience and genomics of psychosis are increasingly *intertwined*. This is to a large extent due to technological convergence (Floridi 2014; Eyre et al. 2017), which I describe in this article as research convergence and data convergence. Along with potential clinical benefits, the translation of biomedical innovation into mental health gives rise to a number of ethical concerns which ought to be systematically addressed.

My argument is composed of two strands. First, I argue that the current translation of biomedical innovation in the context of psychosis requires ethicists to join efforts in order to identify (and respond to) the moral challenges of technological convergence in psychiatry. In other words, I argue that technological convergence in psychiatry is morally problematic—or at least morally significant—and that we should respond to technological convergence with something we might call *ethical convergence*. I suggest that, although extremely important, the sole identification of a number of ethical, legal, and social issues may *not* be sufficient to ensure that we fulfil our duty to promote clinical benefits and minimise potential harms in technology translation. In the case of psychosis, I argue that we should respond to technological convergence by developing an integrated, patient-centred approach focused on the assessment of individual vulnerabilities. In order to do that, I suggest that we (i) integrate insights from several areas of ethics, (ii) translate findings from different areas of bioethics into the mental health context, and (iii) proactively try to anticipate ethical concerns that could derive from future clinical translation.

Second, I argue that the concept of vulnerability might be a useful philosophical tool to accomplish this task. The concept of vulnerability has a long history in research and care ethics and is currently undergoing a thorough theoretical redefinition (Rogers et al. 2012; ten Have 2015). Here, I borrow Florencia Luna’s metaphor of *layers* of vulnerability to describe how the individual assessment of different sources (or degrees) of vulnerability might serve as a common ground for the identification of ethical issues in technological convergence (Luna 2009, 2019). I argue that we can conceptualise potential harms or wrongs to individuals who suffer from psychosis as stemming from different layers of vulnerability. I suggest that a nuanced understanding of vulnerability as it is currently emerging in research and care ethics might help us to achieve the ethical converge, or integration, which is needed to deliver practical solutions to ethical dilemmas in the context of psychosis.

## Psychosis: biomedical innovation and technological convergence

Psychosis is not a discrete diagnostic category. It is an abnormal state of the mind, a set of symptoms characterised by a progressive detachment from reality. Mental disorders which are primarily characterised by psychotic symptoms, such as schizophrenia, are defined as psychotic disorders. Those who suffer from psychosis experience hallucinations, delusions, disorganised thinking and speech, and negative symptoms such as diminished emotional expression and social withdrawal (American Psychiatric Association 2013). Illness aetiology involve at the same time biological, psychological, and social factors (Fusar-Poli et al. 2012). Whilst critiques of the biomedical model of mental illness continue to characterise the scientific debate, novel tools offered by neuroscience and genomics hold promise to unveil the neurobiology of psychosis.

Clinical research on the neurobiology of psychosis has been performed for decades (Ross et al. 2006; Read and Dillon 2013). The novelty of this approach does not lie in the attempt to unveil the neurobiological substrates of psychosis. Rather, the novelty lies in the development of new and more powerful medical technologies through which this attempt is carried out. More specifically, there are two aspects of novelty in recent neurobiological approaches to psychosis: the use of technology originally developed in biomedicine, particularly neuroimaging and next-generation sequencing, and the phenomenon of technological *convergence*. In turn, technological convergence can be described as (i) research convergence or convergence of different research approaches—in this case neuroscience and genomics, as exemplified by the Neuroscience in Psychiatry Network (Inkster et al. 2018) or the UK Biobank project (Elliott et al. 2018)—and (ii) data convergence or convergence of different data sources. Data convergence is evident in recent efforts to translate neurobiological findings into diagnostic tools that use machine learning to enhance diagnosis and prediction of psychosis (Shatte et al. 2019; Young et al. 2016).

Let us explain this further. The neurosciences and genomics of psychosis constitute two distinct domains of investigation. Ethics usually targets one of the two, be it the use of neuroimaging in the context of mental illness (Bluhm et al. 2015) or the application of genomic science to psychiatry (Nuffield Council on Bioethics 1998). However, the two domains are inherently intertwined. Genomic science aims to unfold the molecular processes that govern hereditary patterns leading to neurophysiological and functional abnormalities—correlating with psychopathology—which in turn are the object of clinical neurosciences.

Amid recent developments, the decreasing cost of neuroimaging techniques has rekindled the interest in the neurosciences of psychosis. Magnetic Resonance Imaging (MRI) is used to investigate brain volume and structure in schizophrenia, the most replicated findings being decreased intracranial and total brain volume, along with alterations in grey matter structures (Haijma et al. 2013). Conversely, functional MRI (fMRI) is used to investigate regional brain activity as this reflects disrupted cognitive processes. In addition, molecular imaging techniques such as Positron Emission Tomography (PET), Single Photon Emission Tomography (SPET), and Magnetic Resonance Spectroscopy (MRS) are used to investigate neurotransmitter dysfunction and drug-receptor interaction (McGuire et al. 2008). Thanks to the use of these technologies, abnormal dopaminergic mechanisms have been confirmed to play a central role in psychosis, leading to the proposal of the ‘dopamine hypothesis of schizophrenia–version III’ (Howes and Kapur 2009). The second development that is essential to mention is the introduction of Next-Generation Sequencing (NGS) into psychiatric genomics. This has resulted in the implementation of GWAS on psychosis and schizophrenia. The most interesting findings are the identification of 108 schizophrenia-associated loci contributing to the risk of developing the disorder, along with insights from convergent functional genomics (Schizophrenia Working Group of the Psychiatric Genomics Consortium 2014; Ayalew et al. 2012). As research efforts made possible by the use of NGS progress, new discoveries will likely link our understanding of the genomic variations involved in susceptibility to psychosis with the neurobiological processes associated with illness progression. A clear example of this dynamic is the recent discovery that increased expression of the C4A gene is associated with an increase in *synaptic pruning*, which has been welcomed as a turning point in our understanding of the biology of schizophrenia (Dhindsa and Goldstein 2016).

The convergence of neuroscience and genomics is also evident in the efforts to translate neurobiological findings into clinical care. Integrating genetic, cognitive, and multimodal neuroimaging data could support the classification of clinical populations and may help to identify individuals at high-risk of psychosis (Pettersson-Yeo et al. 2013). The search for markers of psychosis progression has the potential to support diagnosis and benefit treatment options. Possible markers of psychosis progression include, for instance, neuroanatomical markers in high-risk populations and neuro-functional markers in the psychosis prodrome (Koutsouleris et al. 2015; Fusar-Poli et al. 2012). Particularly promising are the attempts to develop tools for psychosis prediction. Recently, Koutsouleris et al. were able to correctly predict transition outcomes in high-risk individuals in 80 per cent of cases using structural MRI data (Koutsouleris et al. 2015). Even more exciting for many researchers is the developing field of psychosis prediction by integration of several data sets via machine learning and narrow artificial intelligence (Gifford et al. 2017; Shatte et al. 2019).

## Technological convergence and ethical, legal, and social issues

### A case study^1^

Tom is 17 years old. He lives with his mother Anna and his father David. David works odd jobs. He has a history of mental health problems and has received a number of diagnosis over the course of the years. Tom has always done very well in school, he likes playing football and going to concerts. However, in the last year Tom has been quite distressed. During a visit with the family doctor, Tom confessed that he has started hearing voices. The doctor referred Tom to the early intervention for psychosis team at the local psychiatric unit. The early intervention team has assigned Tom a care coordinator and offered that Tom attend talking therapy sessions. Meanwhile, a clinical research team is conducting a study within the psychiatric unit. They approach Tom and his family and offer that Tom be included in the research. Over the next 6 months, Tom would have to attend hospital visits once a month for neuroimaging scans. A single blood draw would be performed during the first visit to allow for genetic analysis. In addition, Tom would be given a smartphone that collects behavioural data to monitor his mental health. A smartphone app would help Tom to cope with episodes of voice hearing and allow him to communicate with the research team.

Tom’s story exemplifies the theoretical density which characterises technological convergence in mental health. What moral challenges can we identify in Tom’s story?

Should medical technologies be translated into mental health care? The mere fact that biomedical innovation is being translated into mental health care represents per se a development worthy of ethical scrutiny, given the opposition that psychosocial approaches have traditionally expressed towards the biomedical model of mental illness and its history of involuntary hospital admissions and social control (Read et al. 2013). In other words, one may argue that medical technology should be translated into mental health care *only if* we accept the biomedical model of mental illness. The biomedical model is not universally recognised as valid, considering the complex spectrum of aetiological theories of mental illness. In this article I accept—for the sake of the argument— that the biopsychosocial model of mental illness is valid, and that therefore biomedical innovation has a legitimate role in tackling psychosis.

In recent years, several ethicists have started to identify a number of Ethical, Legal, and Social Issues (ELSI) that characterise the expansion of neuroscience and genomics into mental health. The development of neurobiological approaches to psychosis is situated at the *intersection* of several ethics domains. Referring to the ELSI literature might help us to shed light on the moral challenges that characterise our case study.

First, (neuro) ethicists have started to address the ELSI that arise from the use of neuroimaging in different clinical domains, including psychiatry. Racine and Illes have provided a useful platform for identifying the ethical challenges of advanced neuroimaging research (Racine and Illes 2007). In their comprehensive review, the authors list a number of areas of concern, including management of Incidental Findings (IFs), informed consent, privacy and confidentiality, impact on vulnerable populations, and stigma and discrimination. The links between neuro-essentialist thinking and stigma attached to mental illness play a central role in understanding the societal challenges related to the translation of neurobiology into psychiatry (Haslam 2011). In addition, the use of neuroimaging for diagnosing a psychotic disorder could affect the sense of responsibility and agency of young individuals. Boyce has also noted how the introduction of neuroimaging in psychiatry could result in the excessive adoption of the medical model, potentially affecting clinician-patient relationships and increasing the risks of medicalization and over-diagnosis (Boyce 2009).

Second, psychiatric genetic research might exacerbate ethical concerns traditionally found in genomics. For instance, the question of which results should be communicated to participants is being widely debated given the difficulty of interpreting NGS results, and the potential impact of such information on participants’ life choices. Lázaro-Muñoz et al. argue that additional guidance should be available for evaluating which results should be returned to participants in psychiatric genetic research (Lazaro-Munoz et al. 2018). This issue overlaps with the question of how to manage IFs in psychiatric genomics. In their review of the ethical issues at the intersection of psychiatric genomics and mental health treatment, Kong et al. also mention the connection between genetic essentialism and stigma as a major ethical concern (Kong et al. 2017). In addition, as highlighted by Appelbaum and Benston, the potential development of psychiatric genetic testing renews a number of ethical concerns regarding the translation of psychiatric genomics into the wider domains of general and reproductive health (Appelbaum and Benston 2017).

Third, important legal issues arise at the intersection of research and care. To mention some, the introduction of medical technologies in research on psychosis generates concerns about informed consent. How do researchers obtain informed consent from (young) individuals who suffer from psychosis? How should they? What about individuals who are defined as being at risk of psychosis, but have not (yet) suffered from a psychotic episode (Morris and Heinssen 2014)? In this regard, how will the logic of risk in psychosis prediction interact with the practice of risk assessment and involuntary hospital admission under mental health legislation (Corsico 2019)? Further, GWAS in psychiatry as well as neuroimaging studies generate great amounts of health-related sensitive data. Data handling and sharing will be dependent upon data protection frameworks in different jurisdictions.

In Table 1, I try to list the most relevant ELSI that arise from the convergence of neuroscience and genomics in the context of psychosis. This list does not pretend to be exhaustive. I do not claim that the sole identification of a number of ELSI provides sufficient grounds to address them. Rather, as I explain later, I claim quite the opposite. In the list, I also attempt to identify the most relevant ethical concerns in our case study, Tom’s story.

**Table 1:** ELSI that arise from the convergence of neuroscience and genomics in the context of psychosis

	Neuroscience	Neuroscience and genomics	Genomics
Research	Neuroimaging research governance	– Return of results – Management of Incidental Findings – Lack of immediate clinical utility – Challenges for REC/IRB review	– NGS research governance
Research & care	– Neuro-essentialism – Potential applications in forensic psychiatry	– Informed consent – Impact on identity and agency – Privacy and confidentiality – Data management and sharing	– Genetic essentialism
Clinical/psychiatric care	– Impact on treatment options & compliance	– Stigma & self-stigmatisation – Impact on clinician-patient relationship – Risk assessment and communication – Risk of over-diagnosis – Resource allocation – Impact on families and carers	– Regulation of genetic testing and screening – Potential development of Direct To Consumer psychiatric genetic testing – Risk of genetic discrimination

Tom’s story and the above ELSI review demonstrate that technological convergence in the context of psychosis is situated at the *intersection* of several ethics domains. The same could be said of mental health conditions other than psychosis. Therefore, I believe that we need an epistemological shift in the way we understand the relationships between bioethics and mental health. I do *not* claim that the ELSI approach is not well suited to identify the moral challenges of biomedical innovation in psychiatry. Instead, I argue that the phenomenon of technological convergence may require ethicists to go *beyond* the identification of ELSI in different domains, and to consider joining efforts in what we might call *ethical convergence*, in order to address the moral significance of biomedical innovation in psychiatry. I clarify my argument in the following pages.

## Should ethicists join efforts to address biomedical innovation in the context of psychosis?

As exemplified by Tom’s story, an intricate knot of ELSI characterises the convergence of neuroscience and genomics in the context of psychosis. This knot is situated at the intersection of several ethics domains. How do we untie it?

My argument is meta-ethical in nature. To be more precise, my argument is *not* meta-ethical in the sense of being developed within the scope and aims of (analytic) meta-ethics (Sayre-McCord 2014). I am not attempting to establish the nature of moral claims using the conceptual tools developed within the meta-ethics tradition. Instead, I use the word ‘meta-ethical’ to indicate that my argument is *not only* an ethical argument, as it is primarily located at the epistemological level rather than at the normative level. In this sense, my argument is ‘meta’ ethical as it goes beyond the identification of moral principles to discuss the epistemological relationships amongst different branches of ethical inquiry. The argument goes as follows:

1. Clinicians and researchers have (and, to a certain extent, share) a moral obligation to promote clinical benefits and minimise potential harms in technology translation, notwithstanding the different moral obligations related to their specific professional role.^2^
2. Potential harms can be conceptualised as stemming from the different sources (or *layers*) of vulnerability, which characterise different individuals who suffer from psychosis.
3. The ELSI approach is useful to identify layers of vulnerability and potential harms/wrongs. However, this may not be sufficient. We must integrate insights from different areas of ethics and ELSI research, in order to ensure that different individuals receive the appropriate protection to which they are entitled by virtue of their (degree of) vulnerability.

Point (1) of my argument is grounded in the principles of beneficence and non-maleficence. It serves as a major premise and, as a postulate, it also grounds the ELSI discourse on biomedical innovation in the context of psychosis. For these reasons, I do not think it is necessary to discuss it here. The concept of (layers of) vulnerability is then central to my analysis (Luna 2009). It is an essential philosophical tool that provides some common ground, a blueprint for the integration of different ethical perspectives. Placing the vulnerability of individuals who (may) suffer from psychosis at the core of our ethics discourse might help us to ensure that, in addressing each of the identified concerns, we can establish the appropriate level of protection to which each individual is entitled. I shall argue why I believe that the concept of vulnerability may still be useful in the next section of this article. Before I do that, I wish to justify point (3) of my argument by asking again, how do we untie the intricate knot of ELSI? Let us consider our options.

Option (a) is that we try to establish a new ethics sub discipline. For instance, Cheung has argued that we need a new ethics of psychiatry, which may help us to identify and address the ethical challenges posed by the development of translational neurosciences and by the use of neuro-technology in psychiatry (Cheung 2009). Option (b) is that we keep identifying ELSI arising from neuroscience and genomics, and we then try to provide appropriate solutions in the specific case of psychosis. Lastly, option (c) is that we try to integrate insights from different ethics and ELSI sub disciplines in a patient-centred approach based on the assessment of individual vulnerabilities.

Option (a) sounds promising. However, it may not be currently viable for two reasons. First, we may disagree on whether we need a ‘new ethics of psychiatry’. Even if we could reach a consensus on the matter, establishing a new discipline may prove more difficult than declaring the need for it, whereas some of the ELSI identified require a prompt response. In the 10 years that have passed since Cheung formulated this proposal, neuroethics has developed as a fully independent discipline. However, albeit sometimes overlapping, neuroethics and psychiatric ethics still remain separate, given that the former mainly focuses on neuro-technology and brain conditions and the latter on mental illness (Illes and Sahakian 2011; Sadler et al. 2015). Second, even within a ‘new ethics of psychiatry’ we would still need to provide appropriate solutions in the specific case of psychosis. We would also still need to integrate findings from other ethics sub disciplines, as it would be unrealistic to think that a new discipline could provide novel solutions to all issues addressed by other sub disciplines.

I believe that option (b) best describes current scholarly efforts. Within option (b), we may effectively address the ELSI identified in the case of psychosis, as the growing literature demonstrates. However, option (b) comes with two risks. First, we risk that when addressing each ELSI we may not consider the *interaction* amongst different ELSI. More specifically, as shown in the above review, the ELSI discourse tends to focus on *either* the neuroscience *or* the genomics of psychosis. By limiting our analysis to this approach, we risk losing the bigger picture and forgetting that we are talking about the same individuals, as exemplified in Tom’s story, who must deal with issues of consent, stigma, and neuro and genetic essentialism, only to mention some. In turn, there is a risk that regulation may be driven by only one, or some, of the identified concerns. Second, by not considering the interactions between the ELSI discourse in neuroscience and genomics, we might not be able to provide sufficient grounds to establish whether a specific technology *ought to* be translated in clinical practice.

Option (c) implies that we integrate insights from different ethics sub disciplines in a patient-centred approach focused on the assessment of individual vulnerabilities. It requires that we reflect on what happens, or may happen, to individuals who suffer from psychosis. This would help us to assess not only the impact of neuroscience and genomics in the context of psychosis but also, and more importantly, their convergence and interaction. By adopting option (c) we are prompted to recognise two facts. First, the conduct of clinical research is grounded in the value of scientific knowledge. Yet, this has to be weighed against potential harms and wrongs to research participants. As I describe later in more details, potential harms and wrongs can be conceptualised as stemming from different (individual) layers of vulnerability. Second, the translation of medical technology in psychiatry is grounded in the principle of beneficence, as it aims to ameliorate prevention, diagnosis, and treatment for individuals who suffer from psychosis. However, non-maleficence mandates that people are not exposed to unnecessary risks and harms. In the case of (young) individuals who suffer from psychosis, establishing whether the risks of participating in clinical research are acceptable, or whether treatment options may be beneficial or not, will require that we consider *all* the ELSI involved, as well as their interactions.

For these reasons, I support option (c). At a practical level, I suggest that ethicists should ‘join efforts’ to meet the moral challenges posed by technological convergence in psychiatry, as the case of psychosis appears to demonstrate. At the epistemological level, I believe that we can further specify three *recommendations* which I think are embedded in this proposal.

First, as argued above, we should *integrate* insights from different areas of ethics and ELSI scholarship. While we progress in identifying ELSI arising from the translation of medical technology in psychiatry, adopting an integrated and patient-centred approach will ensure that the particular needs of (young) individuals who suffer from psychosis remain at the core of our ethics reflection. Second, we should *translate* findings from different areas of bioethics into the mental health context. It is of course important to acknowledge that mental health should receive the same level of attention and, proportionally, the same level of resources as physical health. However, in some respect, mental health is qualitatively different from physical health. The impact of psychosis on people’s sense of identity and the cultural understandings of mental illness ought to be taken into consideration (Boydell et al. 2010; Patel et al. 2014). Ethical recommendations drawn in the context of physical health—such as the ones formulated for brain imaging or genomics—must undergo appropriate translation when formulated in context of psychosis. Performing this translation requires that ethical recommendations take at least into account: (i) cultural perceptions of mental health conditions, including stigma; (ii) the peculiarity of caring practices and clinician-patient relationships in psychiatry; (iii) the impact of mental health legislation on regulatory environments. Appropriate translation of ethical recommendations is essential to ensure that they can be properly enacted by the relevant actors involved. Third, we should *proactively seek to anticipate* ethical concerns that may derive from technology translation into clinical care. Technological convergence in the context of psychosis has been, to date, primarily confined to clinical research. However, as I have argued above and as exemplified in our case study, clinical translation is already underway. Whilst medical technologies move from research to care, it is essential that we try to anticipate imminent ethical, legal, and social challenges. The different degrees of vulnerability of psychiatric populations mandate a high level of awareness regarding future clinical developments. The idea of a proactive approach to the ethical evaluation of novel technologies—as opposed to a reactive approach in ethics— is already being discussed regarding neuro-engineering, assistive and rehabilitation technologies (Ienca et al. 2017). Adopting a proactive approach will be important in order to promote the ethical translation of research findings into clinical care.

## Why the concept of vulnerability may (still) be useful

Point (2) of my argument is that “potential harms can be conceptualised as stemming from the different sources (or *layers*) of vulnerability which characterise different individuals who suffer from psychosis”. The assessment of individual vulnerabilities is central to my analysis. I believe that the concept of vulnerability may still be a useful philosophical tool to guide the ethical integration I describe above. Identifying ethical issues in technological convergence means—to a certain extent—identifying clinical and personal benefits which must be weighed against potential harms or wrongs. How does the concept of vulnerability help us to accomplish this task? In what sense are individuals who (may) suffer from psychosis vulnerable?

Vulnerability is a concept with a long history that spans moral philosophy, research ethics, care ethics and feminist ethics (Rogers et al. 2012). It is beyond the scope of this article to provide a comprehensive account of vulnerability theory. Yet it is important to explain why this concept is useful to our analysis.

A common definition of vulnerability—staying close to the etymology of the term—is that being vulnerable means ‘being open to the possibility of being wounded’, or being at risk of harm (Hoffmaster 2006; ten Have 2015). On the one hand, universal accounts of vulnerability recognise that, as embodied beings, *all* humans can be wounded and thus all humans are intrinsically vulnerable. Care is often defined as response to the intrinsic vulnerability that characterises all human beings (Gastmans 2013). Philosophical accounts such as the one proposed by Martha Fineman consider vulnerability a central feature of the human condition which should ground the political discourse around equality (Fineman 2008). On the other hand, the notion of *vulnerable populations* has been used in research ethics to identify groups of people who deserve special protection because of their greater likelihood of being harmed^3^ . This second, population-based account of vulnerability has historically led to the establishment of stronger safeguards for certain groups—among which are people who suffer from mental illness—but also to their unfair exclusion from research (DuBois 2008).

The idea that those who suffer from mental illness are a vulnerable population is present, for instance, in the 2002 Council for International Organizations of Medical Sciences (CIOMS) ethical guidelines for biomedical research or in the EU Clinical Trials Regulation 536/2014 (Bracken-Roche et al. 2017). However, the population-based account of vulnerability has been heavily criticised. Levine et al. have highlighted its stereotyping nature and ineffectiveness in protecting individuals from harm (Levine et al. 2004). Luna has argued that a labelling approach based on the idea of vulnerable populations fails to recognise the ways in which individuals are *rendered* vulnerable by social and relational factors (Luna 2009). More recently, Bracken-Roche et al. have criticised the population-based notion of vulnerability in the case of psychiatric research participants. They argue that such notion is based on stereotypes around the (lack of) decisional capacity of people who suffer from mental illness, which can lead to paternalism and stigmatisation (Bracken-Roche et al. 2016). At the same time, many authors—and among those Luna and Bracken-Roche—argue that the notion of vulnerability ought *not* to be discarded, but revised.

The notion of *layers* of vulnerability developed by Luna (2009, 2019) can help us to understand how individuals who suffer from psychosis are vulnerable, and why this is relevant to the ethical evaluation of biomedical innovation in psychiatry. Individuals who suffer from psychosis are not vulnerable because they belong to the population of the mentally ill. They are not vulnerable *simply* because of their psychosis. Vulnerability is somehow distinct from diagnostic categories, also because (i) diagnostic categories are historical entities which evolve over time (Guloksuz and van Os 2018), and (ii) with reference to the growing field of psychosis prediction, individuals who are *at risk* of psychosis may be recognised as vulnerable in the absence of a specific diagnosis. In this sense, individuals who may suffer from psychosis are not more vulnerable than all other human beings who are at risk of harm because of some form of illness. At the same time, in rejecting a population-based account of vulnerability we must recognise that individuals who experience psychosis may be *rendered* vulnerable by individual and contextual factors. These factors constitute what Luna calls ‘layers’ of vulnerability. Layers of vulnerability do not automatically characterise certain groups. Instead, an individual assessment of different sources, or layers, of vulnerability can serve as a common ground for the identification of ethical issues.

Let us focus on Tom’s story. In what sense is Tom vulnerable? Broadly speaking, Tom is vulnerable because he is in a situation that could benefit him but also increase his likelihood of being harmed. How could Tom be harmed? First, we should consider *individual* factors as a first layer of vulnerability. We can recognise two important individual factors:

1. Tom’s *capacity* to consent to research or treatment: Tom’s decisional capacity is likely to be affected by his age—he is 17—and by his psychotic symptoms. It would be paternalistic to say that Tom lacks capacity only because of his age and mental illness. At the same time, it is important to assess Tom’s decisional capacity *precisely* because his age and mental illness can affect his ability to appreciate what taking part in research might involve.
2. The fact that Tom is unwell and help-seeking: this fact can increase Tom’s chances of being harmed where the duty of care might lose precedence over the duty to produce knowledge. It also establishes different moral obligations for clinicians and researchers.

Second, we should consider *contextual* factors as a second layer of vulnerability. In this sense Luna’s layered account of vulnerability is relational (Luna 2019). We can recognise at least two important sets of contextual factors in Tom’s story:

1. Family dynamics: not only could Tom’s condition affect his family’s relational dynamics. Accessing information on brain processes and genetic predisposition to psychosis could be perceived as either empowering or distressing by different family members.
2. The social context: the social and cultural context can impact on Tom’s likelihood of being harmed and render him vulnerable. Those who suffer from psychosis are often subject to social stigma and discrimination (Yang et al. 2012). In addition, in many jurisdictions people who suffer from mental illness may be subject to coercion and involuntary hospitalisation (de Stefano and Ducci 2008). Whether this may benefit or harm them is debatable. Yet, it clearly limits the extent to which these individuals can exercise their autonomy.

This overview of different sources of vulnerability is not meant to be exhaustive. My argument here is only that a nuanced understanding of the ways in which Tom, and people in a situation similar to Tom’s may be rendered vulnerable can help us to ensure that potential harms are minimised and potential benefits—or occasions to flourish—maximised. In this sense potential harms (or wrongs) to individuals who suffer from psychosis can be conceptualised as stemming from different layers of vulnerability. A layered account of vulnerability can serve as a common ground for the identification of ethical issues in technological convergence, and can be a useful philosophical tool to develop an integrated and patient-centred approach to technology translation.

Luna further rejects the idea of developing fixed *taxonomies* of vulnerability (Luna 2019). Other bioethicists such as Kenneth Kipnis insist on the importance of identifying taxonomies that may be useful to ethical inquiry (Kipnis 2001). I do not wish to enter this debate here. Yet, I wish to highlight three more reasons why a revised notion of vulnerability might help us to integrate the ELSI discourse around the neuroscience and genomics of mental illness.

First, as argued by Henk ten Have, ‘respect for human vulnerability’ is increasingly recognised as an emerging bioethical principle which can ground normative analysis (ten Have 2015). This fact points us to the necessity of rethinking our accounts of vulnerability, but also to the need *not to discard* the very notion of vulnerable individuals. Second, a nuanced and theoretically rich notion of vulnerability can be useful to both research and clinical ethics (Hurst 2008). Such a notion can help us to address ethical issues at the intersection of research and care, and to identify potential harms and benefits arising from technological convergence in psychiatry. Lastly, a layered notion of vulnerability, as the one proposed by Luna, might help us to develop a relational and participatory account of vulnerability in psychiatry. Within a relational account of vulnerability we may recognise that vulnerability is not a feature of certain groups but a *relation* between individual and contextual factors, which may put some people at increased risk of harm. Further, if we wish to find out how Tom—or people in a situation similar to Tom’s—are or may be rendered vulnerable, why not discussing this directly with them? A participatory account of vulnerability highlights that it might be a good strategy to involve directly individuals who experience psychosis in establishing *how* they are or may be rendered vulnerable (Bracken-Roche et al. 2016).

## Conclusions

How could the proposed framework support bioethical inquiry? Let us consider a brief example. Psychosis prediction via machine learning could soon make its way into psychiatric care (Corsico 2019). It will likely be achieved by integrating several data sets including neuroimaging, genomic, and behavioural data. First, in order to address the moral challenges of psychosis prediction via machine learning we must integrate insights from the (neuro) ethics of brain imaging, the ethics of psychiatric genomics, and consider issues of big data governance. This because we ought to ensure that vulnerable individuals are appropriately safeguarded, in research as in clinical care. Second, recommendations formulated in the context of physical health must be translated in the context of mental health to retain their operational validity. We must assess how machine learning will be regulated in different jurisdictions and how mental health legislation will shape regulatory environments. Lastly, it will be important to adopt a proactive approach, as machine learning is still not extensively used in mental health care. Anticipating the ethical challenges that psychosis prediction via machine learning could generate will help us to ensure that individuals receive the protection to which they are entitled by virtue of their (degree of) vulnerability.

Technological convergence is ubiquitous in biomedicine. In this article I have tried to show how, in the case of psychosis, technological convergence takes the form of an attempt to unveil the neurobiology of psychosis with tools offered by neuroscience and genomics. At the intersection of research and care, such attempt is directed towards the development of better ways of predicting, diagnosing, and treating psychotic illness. I have argued that technological convergence in psychiatry is morally problematic. It requires us to start rethinking the uneasy relationships between bioethics and mental health. I have proposed that we direct our attention to the vulnerability which characterises individuals who (may) suffer from mental illness. We should cross traditional boundaries amongst different areas of ethics and promote an integrated approach based on the assessment of individual and contextual sources—or layers—of vulnerability. In other words, I have argued that ethicists should join efforts to respond to the moral challenges of technological convergence in psychiatry. A revised and philosophically rich notion of vulnerability might help us to accomplish this task. Further, I recognise the centrality of patients and service users in assessing the ways in which they could be harmed or helped to flourish by technological convergence.

By doing so we might take a first step towards ensuring that those who suffer from mental illness receive the appropriate protection to which they are entitled. This may imply, for instance, that novel predictive tools are not translated into psychiatry unless there is sufficient evidence for claiming some form of clinical utility. Or it may imply that specific informed consent procedures are put in place when recruiting asymptomatic individuals at risk of psychosis in clinical research involving neuroimaging or genomic procedures. In this article, I did not directly address any of these potential implications. Rather, I have supported the meta-ethical claim that bioethicists, neuroethicists, and legal scholars should join efforts in addressing these developments. Technological convergence requires us to rethink how those of us who suffer from mental illness are or may be rendered vulnerable, and how they can be helped to flourish. In this sense, psychosis is only one occurrence within the spectrum of mental health conditions. Yet, psychosis may provide us with an occasion to reflect on how to ensure that medical technology truly benefits those who experience mental ill health and are at increased risk of harm.

## Footnotes

1 This case study is largely based on a focusing exercise which I used during focus groups within the ELSI-NAPS research study, registration number: research registry 4255, NHS REC reference: 17/NW/0315. The wording of the case study has been slightly modified from the original version in the focusing excercise.

2 Given the fact that I am discussing the use of *medical* technology in mental health, this moral obligation is grounded in the principles of beneficence and non-maleficence (Beauchamp and Childress 2013) which apply—even though in different ways—to clinicians and researchers alike.

3 See the Belmont Report (The National Commission for the Protection of Human Subjects of Biomedical and Behavioral Research 1979).
